# Identifying, exploring and integrating the spiritual dimension in proactive care planning: A mixed methods evaluation of a communication training intervention for multidisciplinary palliative care teams

**DOI:** 10.1177/02692163221122367

**Published:** 2022-10-28

**Authors:** Jacqueline van Meurs, Anne B Wichmann, Patricia van Mierlo, Robert van Dongen, Joep van de Geer, Kris Vissers, Carlo Leget, Yvonne Engels

**Affiliations:** 1 Department of Spiritual and Pastoral Care & Department of Anaesthesiology, Pain and Palliative Medicine, Radboud University Medical Centre, Nijmegen, The Netherlands; 2 Department of Anaesthesiology, Pain and Palliative Medicine, Radboud University Medical Centre, Nijmegen, The Netherlands; 3 Department of Geriatrics & Centre of Supportive and Palliative Care, Rijnstate Arnhem, The Netherlands; 4 Department of Pain Management and Palliative Care, Canisius-Wilhelmina Hospital, Nijmegen, The Netherlands and Department of Anaesthesiology, Pain and Palliative Care, Radboud University Medical Centre, Nijmegen, The Netherlands; 5 Chaplain at Academic Hospice Demeter, Bilthoven and Policy Advisor Spiritual Care in Palliative Care at Agora, The Netherlands; 6 Department of Care and Welfare, University of Humanistic Studies, Utrecht, The Netherlands

**Keywords:** Palliative care, spiritual care, communication healthcare, interactive learning, multidimensional approach, comprehensive analysis

## Abstract

**Background:**

Patients receiving palliative care value attention given to their spiritual needs. However, these needs often remain unexplored as healthcare professionals lack the skills to identify and explore them and to integrate this information into care plans.

**Aim:**

To evaluate the effects of an interactive communication training intervention for palliative care teams in order to identify and explore the spiritual dimension and integrate it in patients’ care plans.

**Design:**

A mixed methods pre-post study, including self-assessment questionnaires, evaluation of videos with simulated consultations (applied competence) and medical record review (implementation).

**Setting/participants:**

Three palliative care teams including nurses (*N* = 21), physicians (*N* = 14) and spiritual caregivers (*N* = 3).

**Results:**

The questionnaires showed an improvement on ‘Patient and family-centred communication’ of the End-of-life professional caregiver survey (+0.37, *p* < 0.01; the 8-item S-EOLC (+0.54, *p* < 0.01) and regarding the Spiritual Care Competence Scale, on the three subscales used (+0.27, *p* < 0.01, +0.29, *p* < 0.01 and +0.32, *p* < 0.01). Video evaluations showed increased attention being paid to patient’s aims and needs. The medical record review showed an increase in anticipation on the non-somatic dimension (OR: 2.2, 95% CI: 1.2–4.3, *p* < 0.05) and, using the Mount Vernon Cancer Network assessment tool, addressing spiritual issues (OR: 10.9, 95% CI: 3.7–39.5, *p* < 0.001).

**Conclusions:**

Our training intervention resulted in increased palliative care professionals’ competence in identifying and exploring patients’ spiritual issues, and their integration in multidimensional proactive palliative care plans. The intervention directly addresses patients’ spiritual concerns and adds value to their palliative care plans.

**What is already known about this topic?**

- Seriously ill patients face uncertainties regarding their future, their deteriorating health and impending death, all affecting their spiritual needs.
- Patients continuously signal their spiritual concerns and value palliative care professionals’ response to these needs.
- Comprehensive evaluation methods are necessary to evaluate the outcome of multi-disciplinary interventions in the spiritual dimension in palliative care and its integration in proactive care planning.

**What this paper adds?**

- The interactive training intervention for palliative care teams, using patient actors, significantly increased palliative care professionals’ awareness and self-assessed skills in identifying and exploring the patients’ spiritual dimension.
- By integrating the spiritual dimension in palliative care plans, handovers and medical record documentation, the training intervention resulted in significant improvements to proactive multidimensional care planning.

**Implications for practice, theory or policy**

- To increase healthcare professionals’ awareness and skills in identifying and exploring their palliative care patients’ spiritual dimension, an interactive training intervention with patient actors is recommended.
- Training healthcare professionals to identify and explore their patients’ spiritual dimension should include the handover, documentation and integration of this information in proactive care plans.

## Introduction

Palliative care patients’ confrontations with deteriorating health, future uncertainties, and facing death all can raise spiritual issues.^1–3^ Leading international organisations rank addressing this spiritual dimension among the most important of all care interventions.^4–6^ Spirituality can be defined as the dynamic dimension of human life that relates to the way persons experience, express and/or seek meaning, purpose and transcendence, and the way they connect to the moment, to self, to others, to nature, to the significant and/or the sacred.^7^

During patient – healthcare professional contacts, patients continuously give signals of their search for meaning and about their spiritual concerns.^8,9^ As their content and meaning can only be identified by multidimensional exploration,^10^ these concerns’ often remain unrecognised, and thus remain unexplored.^3,8,11,12^ Even when noticed, healthcare professionals are frequently unclear about which next steps should be taken.^10^ Essentially, patient signals about their concerns are often overlooked as they fall outside the familiar ‘clinical reasoning’ frame of reference.^13^ Patients appreciate healthcare professionals’ attention to their spiritual needs,^14–18^ and become more empowered to adapt and self-manage than in cases where attention is only given to their physical needs.^17^ Moreover, addressing spiritual issues is important for proactive care planning, and can influence the quality of patients’ lives and care utilisation.^19,20^

Although healthcare professionals commonly receive communication skills training, dedicated training interventions regarding identifying and exploring spiritual issues are scarce.^21^ In cases where healthcare professionals were trained to discuss these issues, they were found to be more competent to meet their patients’ spiritual needs.^22,23^ In the past, these courses mostly focused on spiritual assessment, without training practical (communication) skills during live patient contacts.^24^ A good alternative to using patients in training courses is to use patient actors; this also has shown promising effects on team processes and patient outcomes.^25,26^ In addition, a training intervention that integrated recommendations from a national guideline on existential and spiritual aspects of palliative care gave promising results.^27,28^

However, more comprehensive methods are necessary to evaluate the effects of these interventions; to date their impact has mainly been measured with self-assessment tools.^24^ Therefore, the aim of this study was to comprehensively evaluate the effects of an interactive communication training intervention for multidisciplinary palliative care teams, based on a national interdisciplinary guideline. We hypothesised it would increase identification, exploration and proactive integration of patient signals regarding the spiritual dimension, as well as improve carers’ spiritual care competences and proactive multidimensional care planning.

## Methods

### Design

A mixed methods pre-post study design was used; the SQUIRE guideline for reporting.

### Setting

The training intervention took place in a small theatre.

### Population

Three transmural multidisciplinary palliative care teams working in the south-eastern part of the Netherlands were approached to participate as a team. Transmural care involves close coordination and cooperation across primary and secondary care settings, tailored to a patient’s needs.^29^ The three teams included physicians and nurses involved in palliative care; each team had a spiritual caregiver. Each team member was asked to participate in both training sessions and to complete the pre and post-tests.

### Recruitment

Of each team, two members were contacted about the proposed training intervention; they in-turn informed their colleagues. Subsequently, all team members received an email in which they were asked to confirm their interest in participating in the two training sessions and completing the pre- and post-test.

### Sample

A team was accepted for the intervention once at least 10 team members, of whom at least two were physicians and one a spiritual caregiver gave consent.

### The intervention

An interactive communication training intervention was developed (Supplemental Appendix I) based on the Dutch guideline ‘*Existential and Spiritual Aspects of Palliative Care*’^27^; the intervention was aimed at increasing participants’ skills in identifying and exploring patient signals regarding the spiritual dimension, and to proactively integrate this into healthcare practice.

**Figure 1.**
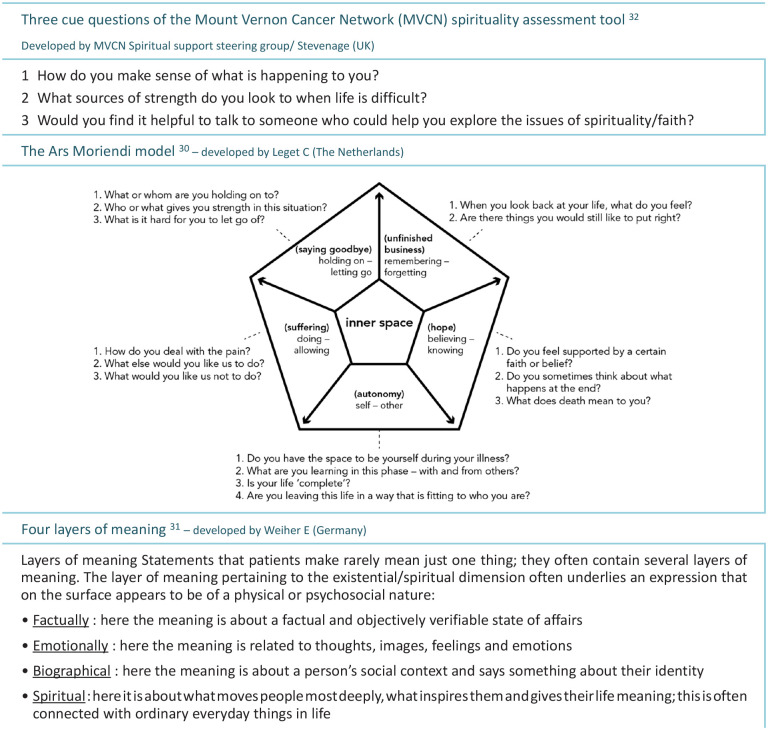
Three (non-validated) tools to explore spiritual aspects of palliative care. Published in: Dutch Guideline ‘Existential and Spiritual Aspects of Palliative Care’.

During the intervention, three (non-validated) tools from this guideline were used to explore spiritual issues in non-crisis situations: the Ars Moriendi model (pp. 87–88),^30^ the ‘Weiher’s Four layers of meaning’ model (pp. 22–23)^31^ and the Mount Vernon Cancer Network assessment tool^32^; (p. 86) (Figure 1).

The intervention was developed by a professional communication trainer, familiar with training healthcare professionals,^33^ incorporating input from YE, ABW and JvM and healthcare professionals. JvM is Researcher and Spiritual Caregiver, ABW is Postdoc in palliative and meaningful healthcare and YE is Professor in meaningful healthcare.

Patient actors simulated palliative patients during the intervention. Groups were trained in two 3.5-h sessions between February and June 2019 in a small theatre.^33^ To enable sufficient interaction with the patient actors, group size was maximised at 15 participants. Each team was trained separately.

The intervention sessions included two lectures: one on proactive palliative care planning^34,35^ (Supplemental Appendix II), and the other on communication. The latter focused on verbal (e.g. ‘*What is the point of suffering like this?*’ or ‘*I no longer recognise myself’*) and non-verbal expressions (symbols of meaning in the patient’s immediate environment, clothing or appearance, as well as the patient’s use of metaphorical language or imagery, e.g. ‘*I feel as if I’m jumping from ice floe to ice floe*’ or ‘*My mind is just as twisted as the infusion’*).^36^ Participants shared cases from their daily practice. Modelled on these examples, simulated healthcare conversations with the patient actors took place. They gave reflections on these conversations, including thoughts, emotions and actions that they would likely take as a patient based on the healthcare professional’s response. The second training session closed with a simulation of transferring the patient’s spiritual dimension information to another healthcare professional.

### Data collection and outcome measures

Following the intervention, participants were asked to indicate their satisfaction with the intervention (0 = not satisfied at all to 10 = extremely satisfied).

Pre- and post-test data a. and b. were collected from the participants 4 weeks before and 4 weeks after the training course. The medical record review (c.) regarding the year prior to and the year following the intervention took place retrospectively (Table 1).

**Table 1.** Time schedule of enrolment, intervention and data collection.

	Study period						
	Month −12	Month −2	Month −1	Month 0	Month 1	Month 2	Month 13
*Enrolment*							
Invitations of teams		X					
Informed consent team members		X					
*Intervention*							
Training session 1				X			
Training session 2					X		
Data collection							
Self-assessment							
12-item subscale patient- and family centred communication of the End-of-life Professional Caregiver Survey (EPCS)			X			X	
8-item Self-Efficacy in End-of-Life Care survey (S-EOLC)			X			X	
Subscales communication, assessment & implementation of spiritual care and referral of the Spiritual Care Competence Scale (SCCS)			X			X	
Applied competence							
Questions regarding videos with simulated patient- healthcare professional consultations			X			X	
Implementation in daily practice							
Retrospectively collected electronic medical record review of deceased patients of the participants	X				X

**a.** **Questionnaire** (self-assessment)

The questionnaire consisted of: (1) the 12-item subscale *patient- and family centred communication* of the End-of-life Professional Caregiver Survey (EPCS),^37^ (2) the 8-item Self-Efficacy in End-of-Life Care survey (S-EOLC)^38^ and (3) the subscales *communication*, *assessment and implementation of spiritual care* and *referral* of the Spiritual Care Competence Scale (SCCS)^39^ (Supplemental Appendix III).

**b.** **Videos with simulated consultations** (applied competence)

As pre- and post-test, the participants observed the same three videos with simulated patient-healthcare professional consultations, modelled on real cases in palliative care. Before recording and editing, essential elements of the composite real-life cases were commented on by a physician and two nurses from a palliative care team and adjusted if required. To facilitate participants’ connection with the cases, only the patient was visible and audible in the final video; the healthcare professional’s words were included as text in the video. Both diversity (age, gender and culture) of the simulated patients and different daily workplaces (home situation, outpatient clinic, clinic) of the target group (nurses, physicians, spiritual caregivers) were considered. Each participant was invited to individually watch these three video consultations online, and to subsequently answer the following questions in an online text box:

*(1) What is this patient most occupied with? How did you notice this?*
*(2) Was there something the healthcare professional omitted or failed to pay attention to during the consultation? If so, what?*
**c.** **Electronic medical record review** (implementation)

Two researchers (AS, ABW) retrospectively collected the documentation on the multidimensionality of care in the Electronic Medical Records of participants working in a clinical setting (four sites) of their deceased patients. The records were verified for documentation of non-somatic (psychological, social and spiritual) dimensions and anticipation on these dimensions, on documentation of patient goals and wishes, and on the use of the Mount Vernon Cancer Network, the Ars Moriendi, and the Weiher tools to explore spiritual aspects of palliative care. Any issues with the collection were discussed with three investigators (ABW, JvM, YE).

A cloud-based valid clinical data management platform (Castor Electronic Data Capture (EDC)) was used for quantitative data management and storage. Qualitative data were collected using Wix and stored in Atlas.ti 8.

### Analysis

During analysis, researchers were blinded for pre- or post-test conditions, and were only unblinded after analysis .

#### Questionnaires

Paired-sampled *t*-tests were conducted to study differences between pre- and post-tests using SPSS version 25. Total scores and scores for questionnaire subscales were analysed, for the whole group as well as for specific healthcare professional groups (nurses and physicians).

#### Videos with simulated consultations

Participants’ written answers to the questions on the simulated consultation videos were coded using directed content analysis.^40^ Categories and codes were predetermined by elements from the Dutch national guideline *Existential and Spiritual aspects of Palliative Care*^27^ practised during the training intervention and discussed during an expert meeting (trustworthiness).^40^ Coding was done independently by two researchers (JvM & ABW). In cases of lack of consensus, a third researcher (YE) was involved (reliability). After unblinding, pre- and post-test results were compared and analysed. Finally, the answers were quantified, using summative content analysis.^40^

#### Retrospective electronic medical record review

Regression analysis was applied to the pre- and post-intervention period, using RStudio Version 1.1.463. A mixed model approach was used, with study centre (hospital) as cluster. Analyses were made on an intention-to treat basis, and on complete case analysis.

### Ethics

The study was performed according to Dutch law and Good Clinical Practice guidelines.^41,42^ The Medical Review Ethics Committee region Arnhem-Nijmegen concluded this study was not subject to the Medical Research Involving Human Subjects Act (case number CMO: 2018-4500). Local ethical committees of the four participating hospitals approved data collection. All data were stored and analysed anonymised.

## Results

### Participants

All three of the transmural palliative care teams approached (24 nurses, 19 physicians, 3 spiritual caregivers in total) gave consent. Non-participation in the intervention was due to personal circumstances, illness and work shifts. The 21 nurses, 14 physicians and 3 spiritual caregivers who participated in both sessions also completed both the pre- and post-measurement (Table 2).

**Table 2.** Characteristics of participants.

*Profession*	
Nurses	
Nurse	9
Nurse practitioner	5
Specialised district nurse	4
Nursing consultant	3
Physicians	
General practitioner	5
Geriatrician	1
Specialist geriatric medicine	4
Internist oncologist	1
Pulmonologist	1
Anaesthesiologist	1
Medical specialist in training	1
Spiritual caregiver	3
Total number	(38)
*Gender*	
Male	8
Female	30
*Age in years* (min-max)	23–63
*Self-estimated expertise in palliative care:* SCALE 0–100 (min-max)	20–80
*Experience in palliative care in years* (min-max)	2–41

### Questionnaires

Participants were very satisfied with the intervention (8.5 on the scale 0–10). Response rates were 90% both in the pre- and post-test. Scores on the subscale *Patient- and family-centred communication* of the EPCS survey increased significantly (+0.37, *p* < 0.01); nurses improved more than physicians. Scores on the S-EOLC also increased significantly (+0.54, *p* < 0.01). Here again, nurses improved more than physicians; at post-test, the nurses reached the physicians’ pre-test values. A significant increase was found (+0.27, *p* < 0.01; +0.29, *p* < 0.01; +0.32, *p* < 0.01) on each of the three subscales of the SCCS. Physicians improved significantly on the sub-scales *Assessment and implementation* and *Referral*, and nurses on all domains (Table 3).

**Table 3.** Differences between pre-test (4 weeks before intervention) and post-test (4 weeks after intervention) on questionnaires.

Questionnaire	Group	∆ (pre- post-test)	95% CI	*p*-Value
EPCS				
*Subscale: Patient- and family-centred communication*	Total group	0.37	−0.52 to −0.23	0.000
	Nurses	0.46	−0.65 to **−**0.28	0.000
	Physicians	0.15	**−**0.37 to 0.07	0.153
S-EOLC				
	Total group	0.54	−0.84 to −0.24	0.001
	Nurses	0.60	−1.04 to **−**0.16	0.011
	Physicians	0.30	**−**0.73 to 0.14	0.160
SCCS				
*Subscale: Communication*	Total group	0.27	−0.45 to −0.09	0.004
	Nurses	0.32	−0.56 to **−**0.09	0.008
	Physicians	0.18	−0.56 to **−**0.19	0.301
*Subscale: Assessment and implementation*	Total group	0.29	−0.42 to −0.16	0.000
	Nurses	0.27	−0.41 to **−**0.12	0.001
	Physicians	0.41	−0.70 to **−**0.12	0.011
*Subscale: Referral*	Total group	0.32	−0.49 to −0.16	0.000
	Nurses	0.27	−0.52 to **−**0.01	0.042
	Physicians	0.42	−0.63 to **−**0.22	0.001
*Overall*	Total group	0.30	−0.42 to −0.18	0.000
	Nurses	0.27	−0.44 to **−**0.11	0.002
	Physicians	0.37	−0.60 to **−**0.14	0.005

Spiritual caregivers are excluded because of their small number (*n* = 3).

### Videos with simulated consultations

*Question 1 ‘What is this patient most occupied with? How did you notice this?’* After the intervention sessions, when the participants answered what in (simulated) consultations patients were most occupied with, their focus was less often (21 vs 6 times) (Table 4) on *their personal predefined agenda*. They gave fewer answers like: ‘does not want to look and think ahead’ or ‘does not accept her illness yet’. Moreover, in post-test the participants’ focus was more often (26 vs 44 times) on the *aims and needs of the patients*, which emerged in answers such as: ‘He wants to make the most of the last part of his life. He still wants to finish his paintings, a triptych’ or ‘Mrs. is mainly occupied with what she can and wants to do*’*. Additionally, when commenting on what the patients were most occupied with, participants’ focus on *death and dying* decreased (28 vs 14 times) while their focus on *the patient’s current life, the here and now* increased (16 vs 20 times). They were also less adamant in interpreting what the patients were most occupied with (47 vs 39 times). Words like ‘trivialise’, ‘avoiding’ or ‘absolutely not’ were used less often. Finally, post-test participants more often asked questions on what the patient was most occupied with (1 vs 6 times).

**Table 4.** Code book of analysis videos.

Question 1: What is this patient most occupied with? How did you notice this?				
Category	Code	Pre	Post	∆ (post – pre)
NOTED				
	Metaphors, symbols	3	0	−3
INTERPRETATION				
	adamant	47	39	−8
	questioning	1	6	**+5**
	seeking meaning	8	10	**+2**
FOCUS				
	nurse or physicians’ personal predefined agenda	21	6	−15
	patients’ aims and needs	26	44	**+18**
	patients’ current life, the here and now	16	20	**+4**
	patients’ death & dying	28	14	−14
Question 2: Was there something the healthcare professional omitted, or failed to pay attention to during the consultation?				
Category	Code	Pre	Post	∆ (post – pre)
NOTED				
	metaphors, symbols	4	13	**+9**
INTERPRETATION				
	adamant	2	2	**0**
	questioning	7	4	−3
	seeking meaning	8	22	**+14**
FOCUS				
	nurse or physicians’ personal predefined agenda	41	19	−22
	patients’ aims and needs	21	18	−3
	patients’ current life, the here and now	7	5	−2
	patients’ death & dying	18	4	−14
EXPLORATION				
	close-ended questions	23	21	−2
	patient-centred way	33	41	**+8**

*Question 2 ‘Was there something the healthcare professional omitted, or failed to pay attention to during the consultation? If so, what?’* After the intervention the participants, when specifying what their colleague healthcare professional in the video failed to pay attention to, more often (33 vs 41 times) suggested exploring in a *patient-centred* way. They would ask ‘what is important in life now, what still gives energy and satisfaction; how and when we can best help’. Moreover, participants less often (41 vs 19 times) focussed on *personal predefined (clinical) agenda’s* and less often (18 vs 4 times) on *death and dying.* Participants more often (8 vs 22 times) pointed out that the healthcare professional on the video had neglected an exploration of the patient’s *meaning seeking*: ‘Medication advice given too quickly; ask what thoughts keep her awake, what would she like to discuss with her husband, what is important to her?’ Moreover, participants more often identified (4 vs 13 times) *symbols of significance in the immediate environment, clothing or appearance, or metaphorical language or imagery* used by the patient: ‘Ask about the meaning of the crucifix she is wearing’ or ‘Ask about ‘the two selves’ she mentions; what does she mean?’.

### Electronic medical record review

A significant increase of 16% was found in the number of notes regarding anticipation on the non-somatic dimension (OR: 2.2, 95% CI: 1.2–4.3, *p* < 0.05) and of 17% regarding identifying and exploring spiritual issues using the Mount Vernon Cancer Network assessment tool (OR: 10.9, 95% CI: 3.7–39.5, *p* < 0.001. No differences were found on notes regarding the use of the Ars Moriendi and Weiher assessment tools (Table 5).

**Table 5.** Documentation in electronic medical record: differences between the year before and the year after the training.

Electronic medical record documentation regarding ..	OR	95% CI	*p*-Value
.. *non-somatic dimension*	0.98	0.37–2.63	0.976
.. *anticipation non-somatic dimension*	2.22	1.18–4.23	0.015
.. *questions Mount Vernon Cancer Network assessment tool*	10.93	3.67–39.54	0.000
.. *referral spiritual caregiver*	0.96	0.23–3.93	0.958
.. *patients’ personal goals*	1.02	0.49–2.12	0.96

## Discussion

### Main findings/results of the study

The effects of an interactive simulation-based communication training intervention in identifying and exploring patients’ spiritual dimension and in integrating this in proactive care planning were assessed in three transmural, multidisciplinary palliative care teams. Differences between pre-test and post-test measurements demonstrated a clear impact on spiritual care competence (self-assessment), an increased attention to patients’ aims and needs combined with a decreased focus on the clinical agenda (applied competence), and an increased use of the Mount Vernon Cancer Network assessment tool and anticipation on the non-somatic dimensions of care in patient records (implementation). We hypothesise that a combination of integration of a national guideline, training in team context, and the variety of training methods including the use of patient actors were responsible for this.

### What this study adds

Effects on self-assessed competence differed between nurses and physicians. Nurses showed a significant increase on all SCCS domains (*communication; assessment and implementation; referral*), physicians only on *assessment and implementation* and *referral*. A comparable Dutch study also found an increase on subscales ‘*assessment and implementation* and *referral*’, but, in contrast to this study, they found no increase on subscale *communication* for both groups.^43^ The intensive practicing with patient actors in our intervention may be the cause of the significantly increased communication skills. We recommend future multidisciplinary training interventions to incorporate different learning needs and goals of various disciplines, and possible effects at team level. The review of the electronic medical record showed that the Mount Vernon Cancer Network spirituality assessment tool is easy to integrate, and thus fits daily care practice well, especially the first question to find out how patients make sense of what is happening to them, or what occupies them most. This finding is in-line with earlier research, and showed the Mount Vernon Cancer Network assessment tool to be most practical and compatible with the medical model.^28^ We recommend that these findings be considered in future education and training interventions, and anticipate that they will also be valuable when training non-expert palliative care providers, plausibly generating even larger effects. Because of their ‘beginners level’, such groups will probably require a more intensive training intervention.

Our findings are relevant for clinical care, as studies have shown that when patients give signals about what occupies them most, these are often left unexplored,^3,8,11,12^ and thus not integrated in oral and documented handover and care planning. Discovering how patients make sense of what is happening to them, or what occupies them most, is fundamental for broader, important concepts such as patient-centred care, total pain, advance care planning, and shared decision making. Therefore, in line with Balboni et al.^19^ we recommend that identifying, exploring and integrating the spiritual dimension is given more attention in curricular education and training interventions. Simulation-based training, the core element of our intervention, is an excellent way to achieve improvements in team functioning,^26^ communication in palliative care,^9^ and notably also in the provision of spiritual care.^44^ Lastly, our findings are relevant for healthcare professionals, as improving communication skills may play a role in reducing burnout among clinicians.^45–47^

### Strengths and limitations

The comprehensive evaluation of the impact of the training intervention is a clear strength. Apart from the analysis of *self-assessed competence* scores, we also measured participants’ *competence to apply* the acquired knowledge and skills and to *implement it in daily care practice*. This profound, unprecedented evaluation of effects is in line with recommendations in recent reviews.^19,24^ Another strength is that the intervention stayed close to daily practice by using real-world case examples from clinical practice, earlier identified as being a key component of spiritual care training.^24^ Moreover, we made innovative use of videos with actual case examples to measure the effects of the training.

A limitations is the absence of a control group. However, as the measured effects all point in the same direction, our findings give a plausible effect of the intervention. There is some sample imbalance: not all disciplines working in palliative care participated. However, the trained teams reflect palliative team composition in the Netherlands, with more nurses than doctors and only one spiritual caregiver. Other healthcare workers such as psychologists or social workers are commonly not part of these teams, but are consulted when required.

The three participating teams all work in the south-eastern part of the Netherlands. However, as they all followed basic and specialised education with national end terms, we do not think this limits generalisability. Moreover, we only measured self-assessed and applied competence effects at one time point, although they are likely to decrease in time, and therefore suggest repeated training interventions. Competence was thereby only measured in the short term (4 weeks before and 4 weeks after the intervention). Moreover, the palliative care professionals’ own (attitude towards) spirituality was not part of the intervention, although an own perspective may cause bias.

## Supplemental Material

sj-pdf-1-pmj-10.1177_02692163221122367 – Supplemental material for Identifying, exploring and integrating the spiritual dimension in proactive care planning: A mixed methods evaluation of a communication training intervention for multidisciplinary palliative care teams Click here for additional data file. Supplemental material, sj-pdf-1-pmj-10.1177_02692163221122367 for Identifying, exploring and integrating the spiritual dimension in proactive care planning: A mixed methods evaluation of a communication training intervention for multidisciplinary palliative care teams by Jacqueline van Meurs, Anne B Wichmann, Patricia van Mierlo, Robert van Dongen, Joep van de Geer, Kris Vissers, Carlo Leget and Yvonne Engels in Palliative Medicine

sj-pdf-2-pmj-10.1177_02692163221122367 – Supplemental material for Identifying, exploring and integrating the spiritual dimension in proactive care planning: A mixed methods evaluation of a communication training intervention for multidisciplinary palliative care teams Click here for additional data file. Supplemental material, sj-pdf-2-pmj-10.1177_02692163221122367 for Identifying, exploring and integrating the spiritual dimension in proactive care planning: A mixed methods evaluation of a communication training intervention for multidisciplinary palliative care teams by Jacqueline van Meurs, Anne B Wichmann, Patricia van Mierlo, Robert van Dongen, Joep van de Geer, Kris Vissers, Carlo Leget and Yvonne Engels in Palliative Medicine

sj-pdf-3-pmj-10.1177_02692163221122367 – Supplemental material for Identifying, exploring and integrating the spiritual dimension in proactive care planning: A mixed methods evaluation of a communication training intervention for multidisciplinary palliative care teams Click here for additional data file. Supplemental material, sj-pdf-3-pmj-10.1177_02692163221122367 for Identifying, exploring and integrating the spiritual dimension in proactive care planning: A mixed methods evaluation of a communication training intervention for multidisciplinary palliative care teams by Jacqueline van Meurs, Anne B Wichmann, Patricia van Mierlo, Robert van Dongen, Joep van de Geer, Kris Vissers, Carlo Leget and Yvonne Engels in Palliative Medicine
