# Supplementary material for: Identifying, exploring and integrating the spiritual dimension in proactive care planning: A mixed methods evaluation of a communication training intervention for multidisciplinary palliative care teams
Source: Palliat Med. 2022 Oct 28;36(10):1493–503. doi: 10.1177/02692163221122367 (PMC9749014; doi:10.1177/02692163221122367)
Supplement: sj-pdf-1-pmj-10.1177_02692163221122367 – Supplemental material for Identifying, exploring and integrating the spiritual dimension in proactive care planning: A mixed methods evaluation of a communication training intervention for multidisciplinary palliative care teams [file sj-pdf-1-pmj-10.1177_02692163221122367.pdf]

Appendix I.

The components of the two training sessions

- **Title of the training:** "Identifying, exploring, proactive; communicating with patients having palliative care needs"
- **Why:** to increase participants' skills in identifying and exploring patient signals regarding the spiritual dimension, and to proactively integrate this into healthcare practice.
- **How:** face to face group sessions; about 50% plenary, about 50% in subgroups of 4 or 5 participants; with the guidance of actors as simulated patients
- **Where:** in a small theatre.
- **When and how much:** two sessions at an interval of 4 to 5 weeks per team, each lasting 3.5 hours.
- **Materials provided:**

  During the first meeting:

  - Updated 'problems square', for anticipatory palliative care planning (Appendix II)
  - workbook including aspects of the Dutch National guideline: 'Existential and Spiritual Aspects of Palliative Care' (1)

  During second meeting:

  PowerPoint presentation on possibilities and challenges of applying the spiritual assessment tools of *Weiher* (2), *Ars Moriendi* (3) and the *Mount Vernon Cancer Network (4)*, from the Dutch National guideline: 'Existential and Spiritual Aspects of Palliative Care' (1)

Detailed content of the training sessions:

Training section	Who	Content	Learning objective
**Training session 1**			
Welcome	Trainer, actors	• The three actors as simulated patients are already in their role without the participants knowing there are training actors: they welcome participants and serve food and drinks; one of them mainly communicates 'emotions', one of them 'facts' and one of them 'opinions'	Introduction to trainer and actors and to the method of training with actors
Introduction	trainer	• Introducing the team<br>• Introduction to the programme<br>• Joint exchange of ideas about thoughts and views of the "spiritual dimension"<br>• Joint exchange of the uniqueness & complementarity of conversations between a patient and the various disciplines present within the palliative team	Follow-up introduction<br>To be informed

Identifying	Trainer + actors	• Exercise with the training actors in further exploring the confrontation at the point of entry: What was heard and seen on welcome?<br>• Converting what is discussed into an exercise with actors and feedback	Training in Identifying
Lecture	Associate professor of timely palliative care	• Practical aspects of proactive palliative care planning with introduction of 'problems square' (5)<br>• Introduction to spiritual assessment tools of Weiher (2) and MVCN (4)	Gathering knowledge of spiritual assessment tools and about proactive palliative care
Identifying (cont.)	trainer + actors	• Short plenary session with actors as simulated patients and use of assessment tools<br>• During the conversation, the 'depth'/'closeness' of a question or the course of a conversation is indicated by physical distance between the simulated patient and the participant<br>• At first the simulated patient moves, at second the trainer asks the participant to move in distance	Experiencing layers in communication (Weiher) and creating closeness in communication
Asking questions (introduction)	trainer	• Jointly explore what types of questions there are<br>• Jointly explore identifying and questioning/exploring	Awareness of the variety of different questions and introduction to types of questions<br>Connecting identifying and exploring
Conversation exercise plenary	actors as simulated patients	• Bringing together identifying and exploring<br>• actors play case as simulated patient and caregiver<br>• Directions from audience, turning in	How to go from identifying and exploration? (plenary)
Conversation exercise subgroups	actors as simulated patients	• Setting a personal sub-learning goal<br>• Split into 2 groups, 1 actor as simulated patient per group<br>• 3 to 4 participants practise a conversation, others observe and give feedback; preferably use of own cases<br>• actor holds feedback round with participants and observers about the experienced	How to go from identifying to exploring? (individual/subgroups)
Recap and homework	trainer	• Short evaluation<br>• Invitation to apply what has been trained in practice (homework assignment): 'write down an intervention on the spiritual dimension from your daily practice'<br>• Sharing a workbook including aspects of the Dutch National guideline: 'Existential and Spiritual Aspects of Palliative Care' (1)	Evaluation

**Training session 2**			
Walk-in/ reception			
Introduction	Trainer	• Explaining the content of programme 2nd meeting<br>• Recalling associations/experiences with the spiritual dimension: are they changed?<br>• Participants write down 1 personal obstacle in entering into conversations on the spiritual dimension in a few minutes; tell about it to a neighbour<br>• Reference to interpretation of spiritual dimension; pointing out upcoming lecture II (spiritual caregiver)	• reactivation of trained in first meeting<br>• Linking the experiences gained in one's own practice with the training course
	Trainer + actors as simulated patients	• Collecting experiences with the homework assignment: 'write down an intervention on the spiritual dimension from your daily practice<br>• Plenary session with actors as simulated patients to practice a part of the situation or conversation described Attention to what is 'new' and brought about a different contact with a patient. Focus on what worked and was experienced as positive<br>• Feed-back from the other participants and the actors	• Linking experiences from one's own practice with the trained
Lecture	Spiritual caregiver	• Lecture on theory and practice of exploring the spiritual dimension within healthcare, including possibilities and challenges of applying the spiritual assessment tools of Weiher (2), Ars Moriendi (3) and the Mount Vernon Cancer Network (4)<br>• Reference to the Dutch National Guideline 'Existential and Spiritual Aspects of Palliative Care'	• Acquiring theoretical knowledge and linking this to one's own practice
Create inner space in conversations	Trainer + actors as simulated patients + lecturers	• Participants are given, in pairs, one of the 5 themes of the Ars Moriendi model, including some example questions<br>• Assignment to explore in 5 groups what the paradigm evokes in relation to one's own practice<br>• Plenary sessions followed with actors on what has been found and thought<br>• Further practice in 2 groups	Awareness and use of inner space (Ars Moriendi) in conversations

The art of asking questions	Trainer	• Serious side of ‘questions’, examples<br>• In discussion with the group, investigate what types of questions we know<br>• Complement with other question types (such as ‘Joker questions’)<br>• Briefly practise with actors as simulated patients	• generate (theoretical) knowledge about the different types of questions
Return to practice	Trainer, actors as simulated patients	• Identifying different roles and discussing which ones overlap: specialist, process facilitator, administrator, coach, neighbour, friend and consider: “What roles do you have as a caregiver?”<br>• 3 roles/3 participants in a row. actor as simulated patient gives game input, what do you do/say from the role taken?	• Role awareness, whether or not to let go of that awareness, being able to switch flexibly between roles, attunement to handover
Hand over	Trainer, actors as simulated patients	• Participants are asked to hand over a case in 1 minute to a colleague<br>• Zoom in on 'handover strategy<br>• Reflection on "transfer strategy"	Integration of the spiritual domain in care planning (handover & electronic medical record)
Wrapping-up	All	• Retrieval, short evaluation	Wrapping up

1. IKNL. Existential and Spiritual Aspects of Palliative Care. Dutch national guideline.: Integraal Kankercentrum Nederland (IKNL); 2018 [2.0:[Available from: https://www.pallialine.nl/uploaded/docs/Zingeving/Existential_and_spiritual_aspects_of_palliative_care_zonder_linken.pdf?u=1SxZX1.
2. Weiher E. Mehr als begleiten: ein neues Profil für die Seelsorge im Raum von Medizin und Pflege: Matthias-Grünewald-Verlag; 2001.
3. Leget C. Art of living, art of dying: spiritual Care for a Good Death: Jessica Kingsley Publishers; 2017.
4. MVCN. Mount Vernon Cancer Network Questionaire. Spiritual support steering group. Final report on spiritual support. Stevenage, UK. 2007.
5. Thoonsen B, Groot M, Verhagen S, van Weel C, Vissers K, Engels Y. Timely identification of palliative patients and anticipatory care planning by GPs: practical application of tools and a training programme. BMC palliative care. 2016;15(1):1-9.
