# Supplementary material for: Identifying, exploring and integrating the spiritual dimension in proactive care planning: A mixed methods evaluation of a communication training intervention for multidisciplinary palliative care teams
Source: Palliat Med. 2022 Oct 28;36(10):1493–503. doi: 10.1177/02692163221122367 (PMC9749014; doi:10.1177/02692163221122367)
Supplement: sj-pdf-2-pmj-10.1177_02692163221122367 – Supplemental material for Identifying, exploring and integrating the spiritual dimension in proactive care planning: A mixed methods evaluation of a communication training intervention for multidisciplinary palliative care teams [file sj-pdf-2-pmj-10.1177_02692163221122367.pdf]

Appendix II: Updated problem square

QUADRANTS for anticipatory palliative care planning

patient name:

patient no:

EXISTENTIAL	
What is going on?	Care plan
Future scenario?	Care plan

How does patient make sense of what is happening? What are sources of strength? Who or what would patient like to have with him/her?

SOCIAL	
What is going on?	Care plan
Future scenario?	Care plan

PSYCHOLOGICAL	
What is going on?	Care plan
Future scenario?	Care plan

PHYSICAL	
What is going on?	Care plan
Future scenario?	Care plan

Radboudumc university medical center

More information?
www.radboudumc.nl/en/research/research-groups/meaningful-healthcare
