# Supplementary material for: Identifying, exploring and integrating the spiritual dimension in proactive care planning: A mixed methods evaluation of a communication training intervention for multidisciplinary palliative care teams
Source: Palliat Med. 2022 Oct 28;36(10):1493–503. doi: 10.1177/02692163221122367 (PMC9749014; doi:10.1177/02692163221122367)
Supplement: sj-pdf-3-pmj-10.1177_02692163221122367 – Supplemental material for Identifying, exploring and integrating the spiritual dimension in proactive care planning: A mixed methods evaluation of a communication training intervention for multidisciplinary palliative care teams [file sj-pdf-3-pmj-10.1177_02692163221122367.pdf]

Appendix III: Questionnaires for intervention participants (pre- and post- measurements)

**EPCS: End-of-life Professional Caregiver Survey**	
Subscale:<br><br>*Patient- and family-centered communication*	1. I am comfortable helping families to accept a poor prognosis<br>2. I am able to set goals for care with patients and families<br>3. I am comfortable talking to patients and families about personal choice and self-determination<br>4. I am comfortable starting and participating in discussions about code status<br>5. I can assist family members and others through the grieving process<br>6. I am able to document the needs and interventions of my patients<br>7. I am comfortable talking with other health care professionals about the care of dying patients<br>8. I am comfortable helping to resolve difficult family conflicts about end-of-life care<br>9. I can recognize impending death (physical changes)<br>10. I know how to use non-drug therapies in management of patients' symptoms<br>11. I am able to address patients' and family members' fears of getting addicted to pain medications<br>12. I encourage patients and families to complete advanced care planning
**S-EOLC: Self-Efficacy in End-of-Life Care survey**	
	1. Discussing the likely course of a life-limiting illness with the patient.<br>2. Discussing the likely course of a life-limiting illness with the patient's family.<br>3. Discussing general issues related to dying and death.<br>4. Having a discussion with the patient about his/her specific concerns about dying and death.<br>5. Having a discussion with the family about their specific concerns about the patient's dying and death.<br>6. Providing emotional support to the family upon bereavement.<br>7. Responding to the patient's question: "How long have I got to live?"<br>8. Responding to the patient's question: "Will there be much suffering or pain?"
**SCCS: Spiritual Care Competence Scale**	
Subscale:<br>*Communication*	1. I can listen actively to a patient's 'life story' in relation to his or her illness/handicap<br>2. I have an accepting attitude in my dealings with a patient (concerned, sympathetic, inspiring trust and confidence, empathetic, genuine, sensitive, sincere and personal)
Subscale:	3. I can report orally and/or in writing on a patient's spiritual needs<br>4. I can tailor care to a patient's spiritual needs/problems in consultation with the patient

Assessment and implementation	5. I can tailor care to a patient's spiritual needs/problems through multidisciplinary consultation<br>6. I can record the nursing component of a patient's spiritual care in the nursing plan<br>7. I can report in writing on a patient's spiritual functioning<br>8. I can report orally on a patient's spiritual functioning
Subscale:<br>*Referral*	9. I can effectively assign care for a patient's spiritual needs to another care<br>10. provider/care worker/care discipline<br>11. At the request of a patient with spiritual needs, I can in a timely and effective manner refer him or her to another care worker (e.g. a chaplain/the patient's own priest/imam)<br>12. I know when I should consult a spiritual advisor concerning a patient's spiritual care
